# Identification of MHC-I-Presented Porcine Respiratory and Reproductive Syndrome Virus (PRRSV) Peptides Reveals Immunogenic Epitopes within Several Non-Structural Proteins Recognized by CD8^+^ T Cells

**DOI:** 10.3390/v14091891

**Published:** 2022-08-26

**Authors:** Marlene Mötz, Melissa R. Stas, Sabine E. Hammer, Tereza Duckova, Frederic Fontaine, Alexandra Kiesler, Kerstin Seitz, Andrea Ladinig, André C. Müller, Christiane Riedel, Armin Saalmüller, Till Rümenapf

**Affiliations:** 1 Institute of Virology, Department of Pathobiology, University of Veterinary Medicine Vienna, Veterinärplatz 1, 1210 Vienna, Austria; 2 Clinic for Swine, Department for Farm Animals and Veterinary Public Health, University of Veterinary Medicine Vienna, Veterinärplatz 1, 1210 Vienna, Austria; 3 Institute of Immunology, Department of Pathobiology, University of Veterinary Medicine Vienna, Veterinärplatz 1, 1210 Vienna, Austria; 4 CeMM Research Centre for Molecular Medicine of the Austrian Academy of Sciences, Lazarettgasse 14, 1090 Vienna, Austria

**Keywords:** porcine reproductive and respiratory syndrome virus, PRRSV, Arteriviridae, CD8^+^ T cells, epitopes, swine leukocyte antigen class I, major histocompatibility complex class I, mass spectrometry, immunopeptidomics, restimulation, intracellular cytokine staining

## Abstract

Porcine reproductive and respiratory syndrome virus (PRRSV) is one of the most relevant porcine pathogens worldwide. Active control of the disease relies on modified live virus vaccines (MLVs), as most inactivated vaccines provide very limited protection. Neutralizing antibodies occur late in infection; therefore, CD8^+^ T cells are considered important correlates of protection and are a frequent focus of investigation. Our aim was to identify viral peptides naturally bound by the class I major histocompatibility complex (MHC-I) and to confirm their ability to stimulate CD8^+^ T cells. For this purpose, we immunoprecipitated MHC-I/peptide complexes of PRRSV (strain AUT15-33) -infected cells (SLA-I Lr-Hp 35.0/24 mod) to isolate the viral epitopes and analyzed them with liquid chromatography coupled to tandem mass spectrometry (LC-MS/MS). Furthermore, we employed these identified peptides to stimulate peripheral blood mononuclear cells (PBMCs) of previously PRRSV-infected pigs and measured the PRRSV-specific CD8^+^ T-cell response with an intracellular cytokine staining (ICS). Our data revealed that PRRSV non-structural proteins (NSPs), encoded in open reading frame 1a and 1b (ORF1), present the major source of MHC-I-presented peptides. Additionally, we show that our identified epitopes are able to trigger IFNγ responses *in vitro*. These findings are a basis for understanding the proteasomal degradation of PRRSV proteins, the cellular ability to display them via MHC-I, and their potential to restimulate CD8^+^ T cells.

## 1. Introduction

Porcine respiratory and reproductive syndrome virus (PRRSV) is an enveloped, single-stranded, positive-sense RNA virus within the family *Arteriviridae,* order *Nidovirales*. PRRSV belongs to the genus *Betaarterivirus* that is divided into two subgenera, *Eurpobartevirus* and *Ampobartevirus*. Each subgenus holds a single species: *Betaarterivirus suid 1*, i.e., PRRSV-1, which is mainly prevalent in Europe, and *Betaarterivirus suid 2*, i.e., PRRSV-2, which is predominantly found in North America and Asia [1]. PRRSV strains show a high degree of genetic variation caused by high mutation rate [2,3], lack of RNA proofreading activity of the RNA-dependent RNA polymerase [4], and recombination events [5]. The virus infects cells of the monocyte/macrophage lineage and can be transmitted horizontally and vertically. PRRSV infection can cause a respiratory syndrome in nursery and fattening pigs, affecting lungs and airways, which is associated with fever and lethargy. A reproductive syndrome only occurs after PRRSV infection of pregnant sows, which can result in late-term abortions, mummification of fetuses, and the birth of weak, congenitally infected piglets [6]. The introduction of PRRSV into a farm is associated with high financial losses and makes PRRSV one of the most relevant pathogens in the swine industry [7]. Although the correlates of protection are not fully understood, modified live vaccines (MLVs) are widely used for prophylaxis and metaphylaxis, but they do bear certain limitations and risks, including limited protection from heterologous PRRSV strains [8] and the potential to revert to virulence [9]. Unfortunately, inactivated vaccines have low efficacy and show weak protection to heterologous challenge [10].

PRRSV manipulates the host immune responses at the humoral and cellular level, resulting in immunosuppression and secondary infections [11]. Affected animals display delayed neutralizing antibody responses [12,13] and disrupted IFN production [14], favoring the replication and spread of the virus. PRRSV additionally downregulates MHC-I expression in infected cells [15,16]. In pigs, MHC-I is a molecule encoded by three classical swine leukocyte antigen complex-I (SLA-I) genes (SLA-1/2/3) and is expressed on all nucleated cells [17]. These cell surface proteins bind and present antigens to CD8^+^ T cells to cause cytotoxic responses. Furthermore, the generation of a CD8^+^ T cell memory protects the host from recurring infections. In naïve cells, MHC-I presents endogenous, mostly cytosolic peptides, to signal that they are not infected. Most CD8^+^ T cell epitopes are generated by the immunoproteasome, an ATP-dependent protease complex, which cleaves ubiquitinated proteins. Nevertheless, there are alternative antigen processing pathways, including autophagy [18] and protein cleavage by furin [19] and by signal peptide peptidases [20]. Classical proteasomal-derived peptides are bound and transported into the endoplasmic reticulum (ER) by the ATP-dependent protein complexes called transporters, which are associated with antigen processing-1 and -2 (TAP1/2). Inside the ER, peptides are loaded by the peptide loading complex (PLC) onto the immature MHC-I α-chain. This chain forms a peptide-binding cleft and has the ability to associate with peptides of most frequently nine amino acids (8–12) in length [21,22]. Longer peptides are rarely bound due to steric hindrance. Once the MHC-I molecule is loaded, it attains its mature conformation, dissociates from the PLC and is transported towards the cell surface for antigen presentation to CD8^+^ T cells. These T lymphocytes recognize the antigen-presenting cells as infected and initiate cytolytic processes, including the production and secretion of cytotoxic granules and cytokines, such as IFNγ and TNFα.

CD8^+^ T cells are considered important correlates of protection in PRRSV-infected pigs [23] since antibody responses are often not sufficient to clear the virus. To date, several studies have investigated the immunologic potential of randomly generated or predicted PRRSV-derived peptides [24,25,26]. However, it is not known whether these peptides are synthesized *in vivo*. The immunoproteasome generally cleaves proteins after hydrophobic and basic amino acids [21]. Therefore, it remains to be experimentally determined if certain predicted or randomly produced peptides are biosynthesized in the cell.

To clarify epitope specificity, our aim was to purify PRRSV-derived MHC-I bound peptide fragments from in vitro infected primary porcine alveolar macrophages (PAMs) to further determine peptide sequence identity by liquid chromatography coupled to tandem mass spectrometry (LC-MS/MS). Additionally, we confirmed the ability of these peptides to initiate a CD8^+^ T-cell-specific cytokine response with an in vitro restimulation assay of PBMCs, isolated from PRRSV infected pigs, followed by an intracellular cytokine staining (ICS), and flow cytometry. With this approach, we provide a method for identifying CD8^+^ T-cell epitopes of PRRSV-infected cells and report on the immunologic potential of peptides originating from the non-structural proteins encoded in open reading frame 1 (ORF1).

## 2. Materials and Methods

### 2.1. Porcine Alveolar Macrophages

PAMs were extracted by bronchoalveolar lavage (BAL) from euthanized, specifically PRRSV-free, and non-vaccinated pigs. Briefly, lungs were removed from the thorax and filled with 1–3 L of lukewarm and sterile PBS, the tissue was gently massaged, and BAL fluid was collected in glass bottles. Cells were washed three times with PBS and centrifuged with a Sorvall RC 26 Plus Centrifuge (Du Pont, Wilmington, NC, USA) at 300× *g* for 10 min at 4 °C. The cell pellet was resuspended in DMEM (Biowest, Nuaillé, France) and the cell number determined with a hemocytometer. Then, 1 × 10^8^ cells were aliquoted in 1 mL fetal calf serum (FCS, Corning, New York, USA) + 10% DMSO (Carl Roth, Karlsruhe, Germany) and stored at −150 °C until further use. The viability of each batch of PAMs and the susceptibility towards PRRSV was assessed by titration.

### 2.2. Animals and PBMC Isolation

Heparinized whole blood was collected from twenty-three 4-month old piglets and two 12-month old gilts. The piglet cells were screened with a peptide pool, consisting of an equal amount of all synthetic peptides from Section 2.10, for an IFNγ response with an ICS. Subsequently, two batches of piglet PBMCs responded to the stimuli and were chosen for testing of the single peptides. Piglets were vaccinated once with Ingelvac PRRSFlex^®^ EU (Boehringer Ingelheim Vetmedica GmbH, Rohrdorf, Germany) and 28 days later challenged with PRRSV strain AUT15-33 (GenBank: MT000052.1). Blood was collected 16 days post challenge. Gilts, which had been previously haplotyped and displayed a similar SLA-I background than the PAMs used for peptide isolation, were challenged with PRRSV strain AUT15-33, and three weeks post challenge, samples were obtained. PBMCs were isolated from heparinized whole blood following density gradient centrifugation (Pancoll human, density 1.077 g/mL, PAN-Biotech, Aidenbach, Germany), as previously described [27]. The recovered PBMCs were counted with a cell counter (XP-300 Haematology Analyser, Sysmex, Vienna, Austria) and cryopreserved in RPMI-1640 with stable glutamine with 100 IU/mL penicillin and 0.1 mg/mL streptomycin (all PAN-Biotech), 40% (*v*/*v*) heat-inactivated FCS (GIBCO, Thermo Fisher Scientific, Waltham, MA, USA), and 10% DMSO (Hybri-Max^TM^, Sigma-Aldrich, St. Louis, MO, USA) at −150 °C until further processing.

### 2.3. SLA-I Typing

PAMs used in this study were genotyped for their SLA class I haplotypes by running low-resolution PCR screening assays. Genomic DNA was isolated from 5 × 10^6^ cells with a commercial kit following the manufacturer’s instructions (E.Z.N.A. Tissue DNA Kit, Omega Bio-tek, Inc., Norcross, GA, USA). SLA-I low-resolution haplotypes (Lr-Hp) were identified by a sequence-specific primed PCR-based typing assay (PCR-SSP) to define the animals’ SLA backgrounds at the allele-group level. SLA typing was performed by PCR-SSP with the complete set of typing primers specific for the allele groups of three SLA class I loci (SLA-1, SLA-2, and SLA-3). The criteria and nomenclature used for SLA-I haplotyping were based on those proposed by the international SLA Nomenclature Committee in the IPD-MHC database of suids (www.ebi.ac.uk/ipd/mhc/group/SLA, accessed on 26 August 2022) [22,28].

### 2.4. Virus

PRRSV-1 field isolate AUT15-33 was produced in PAMs. Cells were seeded in PAM medium (DMEM high glucose, Biowest; 10% FCS; 100 U/mL penicillin; 100 μg/mL streptomycin; 5 μg/mL chloramphenicol; 0.25 μg/mL amphotericin B) in a cell culture dish (Sarstedt, Nümbrecht, Germany) and inoculated with virus stock at a MOI of 0.1 for 1 h at room temperature (RT). The virus was removed and medium added to the cells. After 48 h, supernatant was removed and virus titer determined on PAMs with a TCID_50_ assay. Virus stocks were stored at −80 °C until further use.

### 2.5. Infection of PAMs

PAMs of high susceptibility were thawed at 37 °C and transferred into pre-warmed PAM medium. Four replicates with 5 × 10^8^ cells each were seeded in PAM medium in 135 mm cell culture dishes. Additionally, four replicates of mock-infected cells were seeded in the same manner. One hour after seeding, medium was discarded and PRRSV added at a MOI of 0.1. After 1 h of incubation at RT, the supernatant was removed and fresh medium added to the cells. PAMs were incubated at 37 °C and 5% CO_2_. Successful infection of cells was confirmed with an immunofluorescence staining. Briefly, cells were fixed with 4% paraformaldehyde (Carl Roth) for 20 min at 4 °C. Cells were permeabilized with 1% Triton X-100 (Carl Roth) for 5 min at RT. An in-house Cy3 labelled and produced anti-PRRSV-N monoclonal antibody (clone 810) was used to visualize infected cells.

### 2.6. Immunoprecipitation of the MHC-I/Peptide Complex

Eighteen hours post infection (p.i.), the medium was collected, and PAMs were scraped off in PBS and transferred to a 50 mL tube. Cells were washed three times at 300× *g* for 3 min at RT with a Sigma 3–10 centrifuge (Sigma, Osterode am Harz, Germany). The supernatant was discarded and the cell pellet frozen at −80 °C. For cell lysis, RIPA buffer (150 mM NaCl, 50 mM Tris, 1% NP-40, protease inhibitor cocktail for tissue (Carl Roth), pH 7.5) was added to the frozen pellet and incubated over night at 4 °C. The cell lysate was centrifuged with a fixed-angle Mikro 20 centrifuge (Hettich, Kirchlengern, Germany) at 21.382× *g* for 20 min at 4 °C. The pellet was discarded and the supernatant stored at 4 °C until further use. Protein A/G magnetic beads (Thermo Fisher Scientific) were washed three times with RIPA buffer in a magnetic rack. For the precipitation of MHC-I/peptide complexes, the antibody PT85A was used as previously described [29]. PT85A and isotype control (Mouse IgG2a Isotype Control, Invitrogen, Waltham, MA, USA) were diluted in RIPA buffer and incubated with the magnetic beads for 1 h at 4 °C. Beads were washed three times with RIPA buffer and incubated with the centrifuged cell lysate for 1 h at 4 °C. The beads were washed three times with RIPA buffer and three times with IP wash buffer (150 mM NaCl, 50 mM Tris). At last, the MHC-I/peptide complex was eluted from the magnetic beads with 8 M Urea (BioUltra, ≥99%, Sigma Aldrich, St. Louis, MO, USA) at RT. Samples were stored at −20 °C until preparation for LC-MS/MS analysis.

### 2.7. Western Blot

For the Western blot analysis, the MHC-I/peptide complexes were isolated as described in Section 2.6. However, prior to cell lysis, PAMs were labelled with biotin (EZ-Link™ Sulfo-NHS-SS-Biotin, Thermo Fisher Scientific) for the detection of all cell surface proteins. Protein loading dye (6 M urea, 2% SDS, 10% glycerin, 0.01% bromophenol blue, 0.01% phenol red, 62.5 mM tris) was added to the samples and incubated for 5 min at 95 °C. Samples and a pre-stained protein ladder (New England BioLabs, Ipswich, MA, USA) were loaded on a 7.5% SDS gel and separated at 120 V. Proteins were blotted on a nitrocellulose membrane (BioTrace NT Nitrocellulose Transfer Membrane, Pall, New York, NY, USA) at 70 V for 1 h. The membrane was washed with PBS + 0.1% Tween20 (Carl Roth) and blocked with ROTI-Block (Carl Roth) for 1 h. Avidin horseradish peroxidase (HRP) (Thermo Fisher Scientific) was diluted in ROTI-Block (1:30.000), applied to the membrane, and incubated for 1 h. The membrane was washed three times for 10 min with PBS + 0.1% Tween20 and developed with the ECL Prime Western Blot detection reagent (GE Healthcare, Chicago, IL, USA), according to the manufacturer’s instructions. Western blot was imaged with a ChemiDoc MP Imaging System (Bio-Rad Laboratories, Hercules, CA, USA).

### 2.8. Sample Preparation and LC-MS/MS Analysis

Eluted peptides were reduced by incubation with a final concentration of 10 mM dithiothreitol at 56 °C for 1 h. After cooling down to RT, reduced cysteines were alkylated with iodoacetamide at a final concentration of 55 mM for 30 min in the dark. Urea content was diluted down to a concentration of 2 M prior to desalting and concentrating peptides via reversed-phase solid-phase extraction (SPE) using stage tips with two stacked C18 plugs (Empore™, MERCK KgaA, Darmstadt, Germany) [30]. Briefly, samples were acidified by addition of TFA to a final concentration of 1%. Stage tips were washed three times with 100% acetonitrile and equilibrated three times with stage tip buffer (0.4% formic acid, 2% TFA in water) before loading acidified peptide samples. Salts were removed by washing with 100 μL of 0.1% TFA and purified peptides eluted into a fresh HPLC vial with glass insert two times with 50 μL elution buffer (90% acetonitrile, 0.4% formic acid). Finally, eluted peptides were dried in a vacuum concentrator and reconstituted in 10 μL of 0.1% TFA. Label-free 1D-shotgun LC-MS/MS analysis was performed in a data-dependent acquisition (DDA) fashion on an Orbitrap Fusion Lumos mass spectrometer (Thermo Fisher Scientific) coupled to a Dionex Ultimate 3000RSLC nano system (Thermo Fisher Scientific) via nanoflex ion source interface. Samples were loaded onto a trap column (Pepmap 100, 5 μm, 5 × 0.3 mm, Thermo Fisher Scientific) at 10 μL/min using 0.1% TFA. After loading, the trap column was switched in-line with a 50 cm, 75 μm inner diameter analytical column (packed in-house with ReproSil-Pur 120 C18-AQ, 3 μm, Dr. Maisch, Ammerbuch-Entringen, Germany) thermostatted at 50 °C. Mobile-phase A consisted of 0.4% formic acid in water and mobile-phase B of 0.4% formic acid in a mix of 90% acetonitrile and 10% water. The flow rate was set to 230 nL/min and peptides separated applying a 90 min gradient. MS scans were acquired at 300–1200 m/z in the Orbitrap at a resolution of 120,000 (at m/z 200) and an RF lens amplitude of 40%. AGC targeted 4 × 10^5^ ions at maximum 100 ms. A TopN-dependent scan with a cycle time of 3 s set the acquisition of MS2 spectra in the Orbitrap at a resolution of 15,000 using a fixed first mass of m/z 120 and a quadrupole isolation window of 0.8 Da. Quadrupole isolation was enabled, and HCD was applied with a NCE of 30%. The AGC target was set to 1 × 10^4^ with a maximum injection time of 150 ms. Peptide monoisotopic precursor selection (MIPS) was enabled for charge states 2–6 with an intensity threshold set to 5 × 10^4^ and a dynamic exclusion of 20 sec. A single lock mass at m/z 445.120024 was employed [31]. XCalibur version 4.3.73.11 and Tune 3.4.3072.18 were used to operate the instrument.

### 2.9. Data Analysis and Peptide Sequence Identification

Peak lists obtained from MS/MS spectra were identified using X!Tandem (version X! Tandem Vengeance (2015.12.15.2)) and MS Amanda (version 2.0.0.17442). The search was conducted with SearchGUI (version v4.1.1) [32]. Protein identification was performed against a concatenated target/decoy version of the *NCBI Reference Sequences* (*RefSeq*) (0.5%) database considering the following species: AUT15-33 (8 target/16 decoy sequences), *Sus scrofa* (1431 target/2862 decoy sequences). Decoy sequences were created by reversion of target sequences. The following identification settings were used: unspecific cleavage; 10.0 ppm as MS1 and 0.02 Da as MS2 tolerances; fixed modifications: carbamidomethylation of C (+57.021464 Da), variable modifications: oxidation of M (+15.994915 Da). Peptides and proteins were inferred from the spectrum identification results using PeptideShaker (version 2.2.5) [33]. Peptide spectrum matches, peptides, and proteins were validated at a 1.0% false-discovery rate estimated using the decoy hit distribution. The sequence logo was created with IceLogo (version 1.3.8.) using the *Sus scrofa* reference proteome as consensus.

### 2.10. Synthetic Peptides

The LC-MS/MS-identified peptide sequences were sent to ProteoGenix (Schiltigheim, France) for peptide synthesis. Peptides were manufactured with >80% purity and quality-controlled with HPLC and MS. Synthetic peptides PepPRS02, -03, and -08 were reconstituted in one part DMSO (Carl Roth) and two parts H_2_O under sterile conditions. The remaining peptides were reconstituted in one part acetonitrile (≥99.9%, Sigma Aldrich) and two parts H_2_O under sterile conditions. Peptides were stored at −80 °C.

### 2.11. In Vitro Stimulation of PBMCs

All steps were carried out under sterile conditions using a biosafety cabinet. PBMCs were defrosted in culture medium (RPMI 1640 with stable glutamine, 100 IU/mL penicillin, 0.1 mg/mL streptomycin (all PAN-Biotech), 10% FCS (GIBCO)) and centrifuged with a Heraeus Megafuge 40R (Thermo Fisher Scientific) at 400× *g* for 8 min at RT. Cells were counted with a Sysmex cell counter, and 5 × 10^5^ cells per well, in eight replicates (a total of 4 × 10^6^ cells per condition), were seeded into sterile 96-well round-bottom plates (Nerbe plus GmbH & Co. KG, Winsen, Germany). The plates were incubated at 37 °C and 5% CO_2_ for a minimum of 6 h. Stimulation of PBMCs was carried out with 5 μg/mL peptide or DMSO/ACN as a medium control for 17 h at 37 °C and 5% CO_2_. Four hours prior to harvesting cells, 1 μg/mL brefeldin A (BD GolgiPlug™, BD Biosciences, Franklin Lakes, NJ, USA) was added to inhibit cytokine secretion. A cocktail of phorbol 12-myristate 13-acetate (PMA, 50 ng/mL, Sigma Aldrich, St. Louis, MO, USA), ionomycin (500 ng/mL, Sigma-Aldrich), and brefeldin A was added as a positive control for cytokine production four hours before harvesting.

### 2.12. Intracellular Cytokine Staining

For the intracellular cytokine staining, PBMCs were harvested in PBS (without Ca^2+^ and Mg^2+^, PAN-Biotech) with 3% (*v*/*v*) FCS (GIBCO) and washed twice. The cells were transferred into 96-well round-bottom microtiter plates (Greiner Bio-One, Frickenhausen, Germany) and stained in a four-step procedure. Primary antibody mix (Table 1) was added to the stimulated PBMCs and incubated for 20 min at 4 °C. Further, cells were washed twice in PBS for 4 min at 400× *g* and 4 °C. Secondary antibody (Table 1) and the Fixable Viability Dye eFluor 455UV (Thermo Fisher Scientific) were diluted in PBS and incubated with the cells for 20 min at 4 °C. Cells were washed twice as previously described. Next, cells were fixed and permeabilized using the BD Cytofix/Cytoperm kit (BD Biosciences) according to the manufacturer’s instructions. Intracellular cytokines were stained with an antibody mix (Table 1) in permeabilization buffer for 20 min at 4 °C. Two final wash steps were executed before resuspending the PBMCs in permeabilization buffer. A fluorescence minus one (FMO) sample without anti-TNFα monoclonal antibody (mAb) was prepared as a background control.

### 2.13. Flow Cytometry

The CytoFLEX LX (Beckman Coulter GmbH, Krefeld, Germany) flow cytometer equipped with six lasers (355, 405, 488, 561, 638, and 808 nm) and a plate loader was used for the analysis of the stained samples. The VersaComp Antibody Capture kit (Beckman Coulter) was used to set-up single stains, following the manufacturer’s instructions, in order to calculate the compensation values using the CytExpert software version 2.4 (Beckman Coulter). For all samples, 1 × 10^6^ lymphocytes were recorded in total. Flow cytometry data were processed using FlowJo software version 10.8.1 (BD Biosciences).

## 3. Results

### 3.1. Isolation of MHC-I/Peptide Complexes by Immunoprecipitation

**Figure 1:**
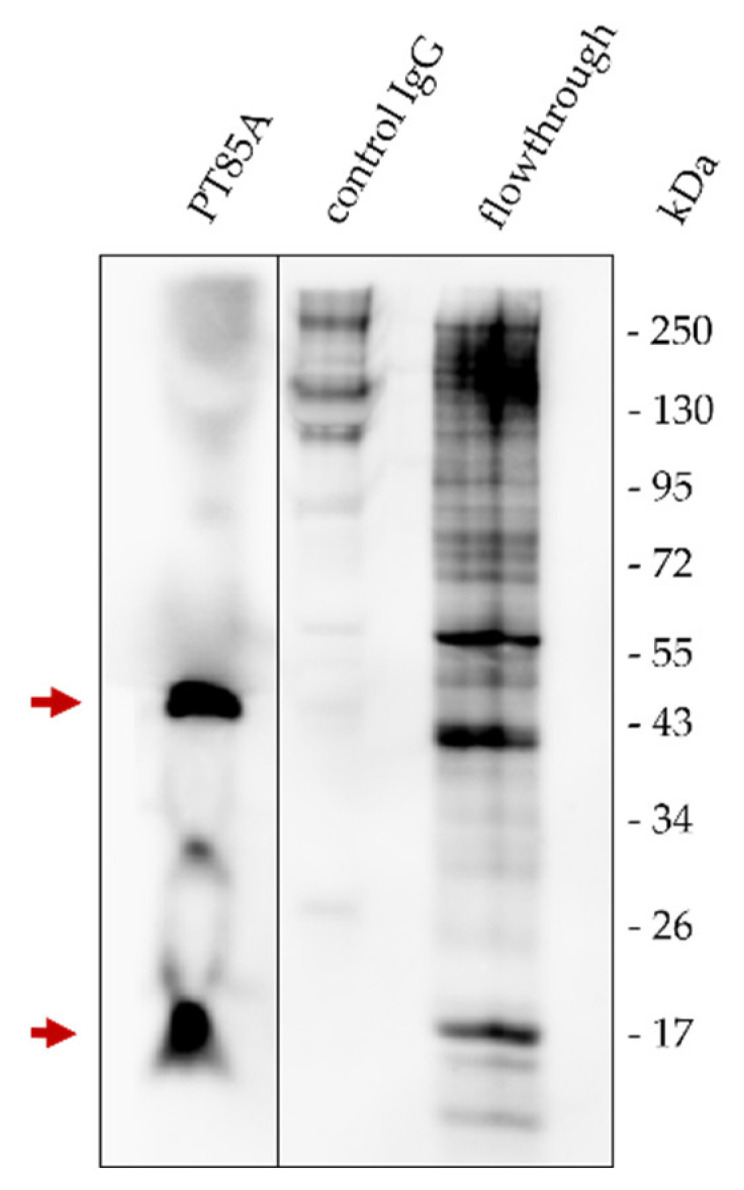
Western blot of isolated MHC-I molecules from porcine alveolar macrophages (PAMs). Cell surface proteins were immunoprecipitated with mAb PT85A linked to protein A/G magnetic beads. A mouse IgG2a isotype control was used to confirm the absence of isotype-specific binding. After running samples on a SDS-PAGE and blotting onto a nitrocellulose membrane, biotin was stained with avidin-HRP. The upper arrow indicates the MHC-I alpha chain at 45 kDa, which harbors the peptide binding groove, and the lower arrow β2-microglobulin at 12 kDa.

Biotinylated PAM lysates were immunoprecipitated with protein A/G beads linked to the anti-MHC-I antibody PT85A. Eluates were analyzed with an SDS-PAGE followed by a Western blot. Detection of biotin-labelled cell surface proteins with avidin-HRP confirmed the successful isolation of MHC-I α-chains at 45 kDa and β2-microglobulin molecules at 12 kDa, without major contaminations with other cellular proteins (Figure 1). The immunoprecipitation with a control IgG did not show isotype-specific binding towards proteins of the cell lysates. After the establishment of the immunoprecipitation protocol, samples for LC-MS/MS analysis were prepared without biotinylation to avoid analyte loss by additional washing steps.

### 3.2. Peptides Originating from PRRSV Non-Structural Proteins Are Displayed by MHC-I

**Figure 2:**
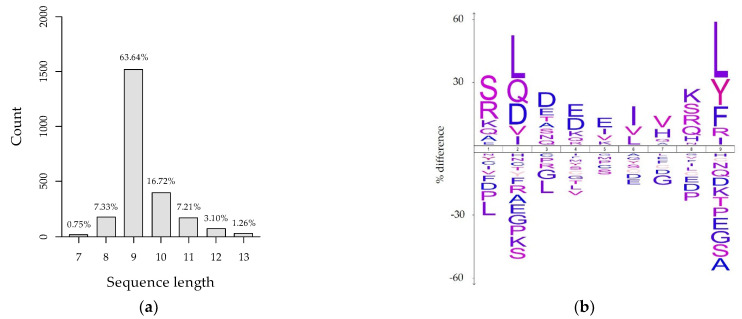
Length and sequence conservation of LC-MS/MS identified peptides from mock-infected PAMs searched against the *Sus scrofa* proteome. (**a**) Peptide sequence length (amino acids) of MHC-I-bound porcine peptides. Percentages of total isolated peptides are displayed above the bars. (**b**) Sequence logo of 9-mer peptides, depicting percent differences of amino acid frequencies from the *Sus scrofa* proteome as a reference.

PAMs were infected with the PRRSV strain AUT15-33 at an MOI of 0.1. Additionally, another batch was mock-infected. From both naïve and infected cells, four technical replicates were produced. Since several studies, including our own unpublished results, demonstrate that MHC-I is downregulated upon PRRSV infection [15,16], cells were harvested at 18 h p.i. to ensure maximum sample yield. Successful infection of cells was confirmed in a small, separate cell culture dish by immunofluorescence (data not shown). After harvesting, the MHC-I/peptide complexes were isolated by immunoprecipitation. Eluted samples were analyzed by LC-MS/MS, and the obtained MS data searched against the AUT15-33 and *Sus scrofa* proteome. Database research of samples from naïve PAMs that were matched to the *Sus scrofa* proteome revealed a total of 2387 identified peptide groups, with peptides between 7 and 13 amino acids in length. The vast majority (63.64%) of those are nonamers (Figure 2a). These findings are in line with the literature, which demonstrates that most MHC-I bound peptides have a sequence length of nine amino acids [34]. Furthermore, the epitope anchor residues are known to preferentially be hydrophobic or basic amino acids [21]. Our data confirms a conservation of hydrophobic amino acids at the anchor residues (position 2 and 9) of 9-mer peptides (Figure 2b).

**Figure 3:**
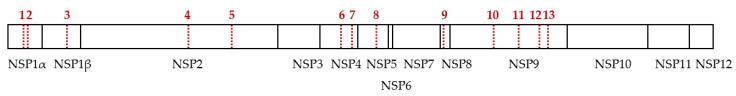
Position of identified MHC-I-bound peptides (dashed red lines) from infected PAMs within the PRRSV ORF1 polyprotein. Numbers above the red dashed lines correspond with Table 2. NSP, non-structural protein.

Notably, the peptide spectrum matches obtained from MHC-I-bound peptide isolates of PRRSV infected PAMs—when searched against the AUT15-33 proteome—gave the most confident hits to proteins within ORF1, which codes for the virus’ non-structural proteins (NSPs, Table 2, Figure 3). Seven peptides were matched to four of the viral proteases—NSP1α, NSP1β, NSP2, and NSP4. One peptide originates from the transmembrane protein NSP5 and one from NSP8. Four peptides were matched to NSP9, the viral RNA-dependent RNA polymerase. To confirm that the LC-MS/MS-obtained viral peptide sequences are also PRRSV-specific CD8^+^ T-cell epitopes, an evaluation of their immunologic potential was conducted.

### 3.3. MHC-I-Bound PRRSV Peptides Elicit a CD8^+^ T-Cell-Specific IFNγ Response

**Figure 4:**
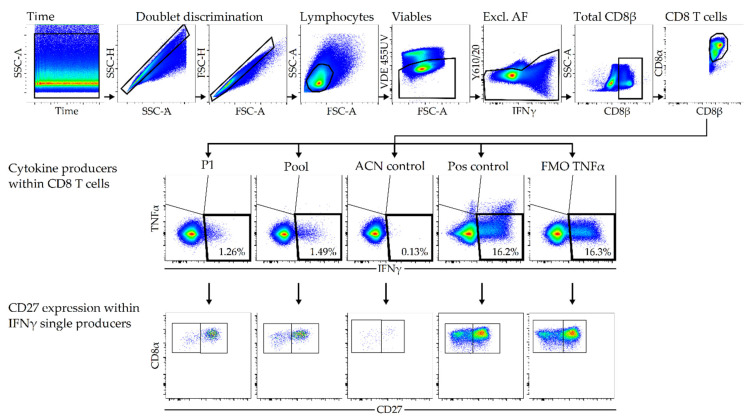
Gating strategy to assess PRRSV-peptide-specific CD8^+^ T-cell responses. Thawed porcine PBMCs were stimulated in vitro with the identified PRRSV-peptides (5 μg/mL), ACN/DMSO, or PMA/ionomycin. Cells were harvested, stained in a four-step procedure and analyzed by flow cytometry. For all samples, a time gate and a double-doublet discrimination (SSC-H vs. SSC-A and FSC-H vs. FSC-A) was applied. Lymphocytes were gated based on their light scatter properties (FSC-A vs. SSC-A); viable cells were selected based on their staining with the Fixable Viability Dye eFluor 455UV and the use of an empty channel—bandpass filter Y610/20—allowed for the exclusion of cells with autofluorescent signal. Thereafter, total CD8^+^ T cells were gated on and were further analyzed for their co-expression of CD8β. These cells were analyzed for their expression of IFNγ and TNFα. The middle panel shows the cytokine production in response to peptide 1 (P1), peptide pool, negative control, positive control, and FMO control. The lower panel shows CD27 expression of IFNγ-producing CD8^+^ T cells in response to the stimuli. Representative pseudocolor plots from one animal are shown.

The thirteen most confident hits for PRRSV ORF1-derived peptides and two *Sus scrofa*-derived peptides (C1: ELNDRFANY, C2: KLRDLEDSL) were synthesized and their immunogenic potential assessed with an in vitro restimulation of PBMCs, followed by an ICS. We preferred an ICS over the classical ELISPOT assay due to its lower background, higher sensitivity, and ability to sort for CD8^+^ T-cell specifically. PBMCs from our biobank of 23 randomly chosen piglets were screened with a pool of 13 LC-MS/MS-identified PRRSV peptides (data not shown). Our screening revealed two responders among the piglets, displaying an elevated IFNγ production compared to the negative controls. To shed light on the piglets’ MHC-I background, the responder PBMCs and the PAMs used for the isolation of MHC-I peptides were SLA-I haplotyped (Table 3). The low-resolution haplotypes (Lr-Hp) revealed that all PBMCs used in this study share a same allele with the PAMs. Further, the four batches of PBMCs were used to assess the immunologic potential of the 13 individual LC-MS/MS-identified PRRSV peptides (P1–P13), the peptide pool, and two endogenous porcine peptides (C1 and C2) in an in vitro restimulation assay. Additionally, a negative control (ACN/DMSO) for negative background activation and a positive or activation control (PMA/ionomycin) were employed. Cytokine production by CD8^+^ T cells and CD27 expression in response to the different stimuli were evaluated via ICS, followed by a flow cytometric analysis. The gating strategy to assess PRRSV-peptide-specific CD8^+^ cytokine responses and CD27 expression is described in Figure 4.

**Figure 5:**
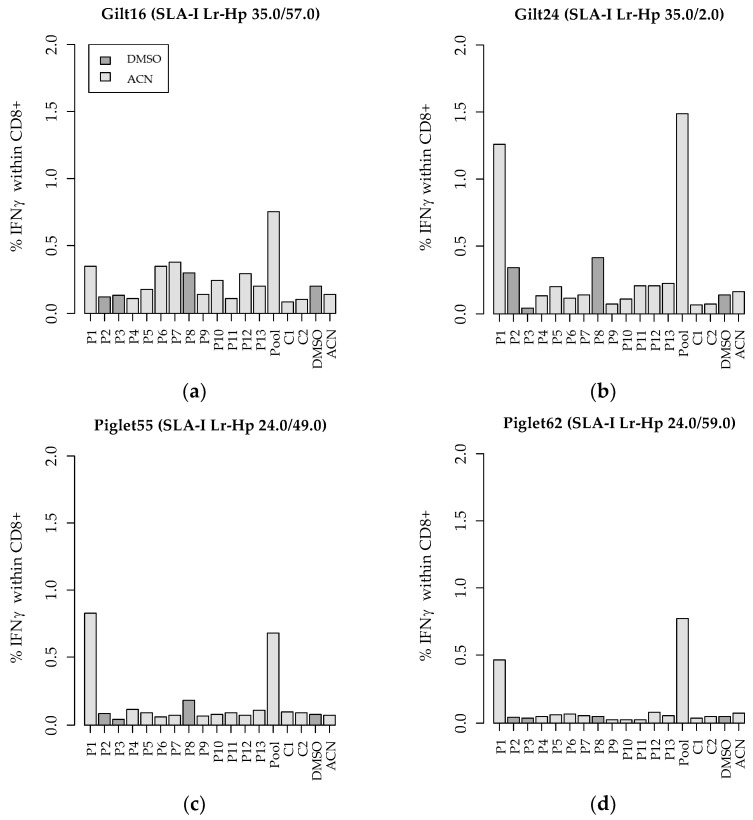
IFNγ-producing cells within the total CD8^+^ T-cell population after restimulation of PBMCs with MHC-I-bound porcine and PRRSV-derived peptides. PBMCs were isolated from two gilts (**a**,**b**) and two piglets (**c**,**d**). P, PRRSV-derived peptides; C, control peptides from naïve cells; DMSO and CAN, medium control; Pool, pool of all 13 PRRSV peptides. SLA-I low-resolution haplotypes (Lr-Hp) of the donor animals are indicated above each graph.

Our analysis revealed that the peptide pool, consisting of equal amounts of all 13 identified MHC-I-bound PRRSV peptides, triggered a CD8^+^ T-cell-specific IFNγ response in all four animals, which was 5.4 to 10.5 times higher than the negative controls (Figure 5). Between 0.69% and 1.49% of the total CD8^+^ T-cell population produced antigen-specific IFNγ after restimulation with the peptide pool. There was no notable rise in TNFα or IFNγ/TNFα co-producing CD8^+^ cells. Notably, peptide 1 (P1) showed the strongest IFNγ response in all animals, which was between 0.36% and 1.26% of the total CD8^+^ T-cell population. MHC-I-bound porcine peptides (C1 and C2) isolated from naïve cells did not trigger the production of IFNγ, confirming their endogenous properties. Additionally, we employed a staining for CD27, a marker for naïve T cells. During differentiation towards an effector (memory) phenotype, CD27 expression is depleted [34]. In our study, the restimulation with the peptide pool and the single peptides exhibited a higher frequency of naïve or CD27^+^CD8^+^ T cells compared to the positive control, PMA/ionomycin, which is a potent stimulus (Figure 4). Between 31.7% and 70.1% of the PMA/ionomycin-stimulated cells, between 43.4% and 73.0% of the cells stimulated with the peptide pool, and between 60.7% and 88.9% of the P1-restimulated cells belonged to this naïve population. The amount of CD27^−^ CD8^+^ cells, which represent a terminally differentiated T-cell phenotype, is the lowest in PBMCs restimulated with P1 compared to the restimulation with the peptide pool and the positive controls. This lets us conclude that the individual peptides are weaker stimulants for T-cell differentiation than pooled peptides.

## 4. Discussion

CD8^+^ T-cell responses are important correlates of protection in PRRSV-infected animals due to their ability to identify and eliminate infected cells. It has been shown that PRRSV-induced neutralizing antibody responses are delayed [13], strain specific [35,36], and often not fully capable of neutralizing the virus [36]. Therefore, the study of the activation of CD8^+^ T-cell effector responses is a crucial task in understanding host responses after PRRSV infections. Additionally, these findings are important for optimizing vaccinations and therapeutic efforts. To understand which viral antigens contribute to eliciting CD8^+^ T-cell responses, immunogenic MHC-I-bound peptides have to be identified.

To date, several studies have been investigating PRRSV epitopes by cross-presentation of either predicted or randomly produced viral peptides or peptide libraries. Wang et al. designed overlapping peptides from PRRSV membrane proteins and identified four immunodominant epitopes. Since all our ICS analyzed PRRSV-specific, MHC-I-bound peptides derived from NSPs, there is no overlap of peptide sequences with this study. Pan et al. [24] predicted nine PRRSV epitopes with NetMHCpan 4.0 [37], of which three were derived from NSPs. One of those peptides elicited an IFNγ response in their analysis but was also not identified in our screen. Chung et al. [38] created an overlapping peptide library of all PRRSV ORFs and evaluated their immunogenic potential by restimulating PBMCs. They identified immunogenic peptides within NSP1α, NSP2, and NSP4 using an ELISPOT assay. One 20-mer within NSP4 is covering the sequence of our identified peptide 7. Another immunogenic 20-mer is partly overlapping with our PRRSV peptides 1 and 2. Parida et al. [39] investigated the stimulation potential of PBMCs with overlapping peptides from PRRSV NSP9 and NSP10 using a proliferation assay in combination with an ELISPOT. They were able to identify immunogenic peptides, but none of them overlap with our analyzed peptide sequences. In addition, Burgara-Estrella et al. [40] and Mokhtar et al. [41] screened PRRSV peptide libraries by ELISPOT, but we could not find an overlap of peptide sequences with the epitopes that we used for our in vitro stimulation experiments. The varying SLA-I backgrounds of the pigs used in the different studies might be an explanation for the non-overlapping peptide sequences. Furthermore, the diversity of identified CD8^+^ T-cell-stimulating epitopes in the literature lets us conclude that there is a much broader PRRSV epitope repertoire than already reported. Nevertheless, the predicted or randomly generated epitopes from these studies are hypothetical, and it is not clear whether they are actually generated *in vivo*. The immunoproteasome has certain patterns of hydrolyzing proteins [21,42], and therefore, only a limited number of these peptides might naturally occur.

With our study, which is based on methods from a pioneer study of the 1990s [43], we provide, to our knowledge, the first sequences of MHC-I-bound peptides that have been directly isolated from cells infected with PRRSV *in vitro*. These peptides are products of ubiquitin-mediated degradation of the immunoproteasome and presented by MHC-I molecules on the cell surface of PAMs. After isolation of the MHC-I/peptide complexes by immunoprecipitation, we analyzed the bound peptides with LC-MS/MS. Our data confirm the successful isolation of porcine endogenous and PRRSV MHC-I-bound peptides. The LC-MS/MS turned out to be the bottleneck of the study since immunopeptidomic analyses are not well-established in most mass spectrometry facilities. The vast majority of peptide sequences from naïve cells, obtained by peptide-spectrum matching with the *Sus scrofa* genome, were 9-mers, which possess hydrophobic MHC-I anchor residues. Furthermore, databank research with the PRRSV proteome of LC-MS/MS-obtained mass spectra from infected cells revealed that the most confident sequences originate from several NSPs of ORF1. These proteins are located in the cytoplasm and are plausible targets for ubiquitin-mediated proteasomal degradation. Interestingly, no confident peptide-spectrum matches were obtained for the structural proteins encoded by ORF 2–7 although they are present in a several-fold molar excess over ORF1-encoded proteins. This is plausible for the four glycoproteins, as they reside in the secretory pathway. However, we would have expected peptides derived from the strongly expressed ORF7 encoded nucleocapsid (N) protein, which is cytosolic. Our data represent only a preliminary glance at PRRSV-derived MHC-I bound peptides, and further in-depth studies have to be conducted to validate and expand these data. A caveat of this study is the heterogeneity of the SLA-class I haplotypes that diverge between peptide isolation and challenge models. There is a large number of known SLA-I haplotypes with altering frequencies in the pig population, and an inbred line would be favorable. Unfortunately, for our study, we did not have access to inbred pigs. Nevertheless, the gain of the genetic heterogeneity shows that our peptides might have the potential of stimulating different SLA-I haplotypes.

To assess whether the determined peptides from ORF1 are able to trigger an IFNγ response of CD8^+^ T cells, we investigated the immunologic responses towards these potential epitopes in a small-scale pilot study. IFNγ production is an important correlate of protection after PRRSV infection to regulate antiviral immune responses [22,44]. In order to investigate whether our LC-MS/MS-identified peptides possess the ability to trigger a PRRSV-specific IFNγ CD8^+^ T-cell response, we restimulated PBMCs, which were isolated from PRRSV-challenged pigs, in an in vitro assay. To detect and measure cytokine production, we performed an intracellular cytokine staining (ICS) followed by flow cytometry. The use of an IFNγ ELISPOT was omitted because of its poorer sensitivity and the inability to assess cytokine production at the single-cell level. Our experiments revealed the presence of IFNγ-producing CD8^+^ T cells upon restimulation with PRRSV peptides. Additionally, the ICS panel included a CD27 staining. Within the porcine CD8^+^ T-cell population, CD27 expression has previously been described as a marker for the naïve phenotype and a lack of CD27 expression as an effector (memory) phenotype [34]. Our results show a lower amount of CD8^+^CD27^−^ T cells upon restimulation with the PRRSV single peptides or peptide pool compared to the positive control. This means there is a larger population of naïve than terminally differentiated CD8^+^ T cells upon stimulation with PRRSV-specific MHC-I peptides compared to the positive control. We hypothesize that this could be due to the short incubation time of the PBMCs with the peptides and/or the relatively weak restimulation potential of our PRRSV-derived single peptides in comparison to the positive control, which is a strong stimulus.

All gilts and piglets, which were the donors of the PBMCs used for our in vitro restimulation experiments, possessed a similar SLA-I background as the PAMs used for the isolation of the MHC-I-bound PRRSV-specific peptides. A model using inbred pigs would be the ideal setting, but due to limited access and animal welfare reasons, cells for our studies were used from pre-existing cell banks. All of the cells used in this study share at least one Lr-Hp but have a different second set of SLA-I alleles. Therefore, we can hypothesize that the immunogenic PRRSV-derived peptides are not necessarily confined to a specific SLA-I haplotype but might have the potential of stimulating others, too. More research is needed to verify this hypothesis.

**Figure 6:**
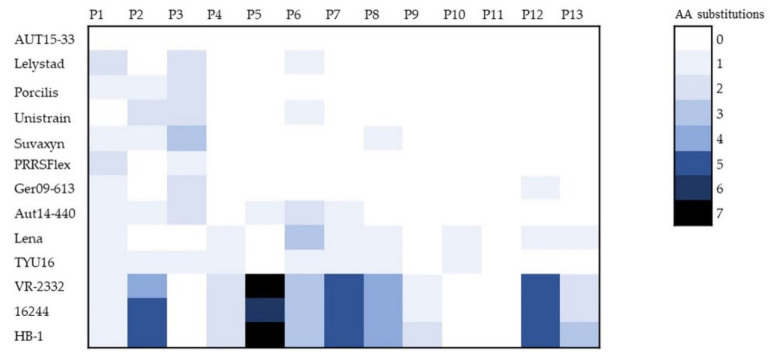
Heat map illustrating conservation of isolated AUT15-33 peptides compared to other PRRSV vaccine strains (94881: Ingelvac PRRSFLEX EU; DV: Porcilis PRRS, MSD; Amervac: Unistrain PRRS, HIPRA; 96V198: Suvaxyn PRRS MLV, Zoetis), PRRSV-1, and PRRSV-2 (VR-2332, 16244, HB-1) isolates. Numbers of amino acid substitutions are coded according to the color key. P, peptide; AA, amino acid.

One limitation in the development of cross-protective PRRSV vaccines is the high mutation rate and occurrence of highly divergent strains in the field. Nevertheless, the viral NSPs have functional constraints limiting variability, while the structural proteins show a lower degree of conservation. Consequently, it can be expected that CD8^+^ T-cell-stimulating epitopes derived from NSPs possess the potential to generate immune responses to multiple PRRSV strains. Our identified epitopes are especially conserved among PRRSV strains used for MLV vaccines, PRRSV-1 strains, and also partly in PRRSV-2 isolates (Figure 6). This could be of importance for the redesign of existing vaccines to make them more efficient and cross-protective against heterologous PRRSV strains. Furthermore, our preliminarily identified PRRSV epitopes could be first candidates for being included in novel, rationally designed mRNA vaccines or vector vaccines.

Our LC-MS/MS- and ICS-based identification method is a straightforward way to identify additional PRRSV-specific CD8^+^ T-cell epitopes. To explore the PRRSV immunopeptidome further, follow-up studies investigating different SLA-I haplotypes and different PRRSV strains have to be conducted to gain a better insight into proteasomal processing, MHC-I presentation, and CD8^+^ T-cell restimulation in PRRSV-infected animals. Additionally, this identification method can be applied to investigate the immunopeptidome of other intracellular pathogens.

## 5. Conclusions

We have established a method for identifying MHC-I-displayed CD8^+^ T-cell epitopes from PRRSV-infected PAMs with an LC-MS/MS-based assay. Further, we confirmed the immunogenicity of some identified peptides by an in vitro PBMC restimulation assay, ICS staining, and flow cytometry measurement. Peptides originating from several PRRSV NSPs were shown to elicit an IFNγ response and are strong candidates for immunization efforts. Further CD8^+^ T-cell epitopes from multiple PRRSV strains and cells with different SLA-I backgrounds must be identified and their immunologic potential investigated in the future to deepen the understanding of ubiquitin-mediated proteasomal degradation and presentation of PRRSV protein-derived peptides to CD8^+^ T cells.

## Figures and Tables

**Table 1:** List of antibodies used in the ICS panel.

Marker	Clone	Isotype	Fluorophore	Labelling	Source
**Surface antigens**					
CD8α	76-2-11	IgG2a	PerCPerfluor 710	Indirect ^A^	In house
CD27	b30c7	IgG1	Alexa fluor 647	Direct	In house
CD8β	PPT23	IgG1	Alexa fluor 488	Direct	In house
**Intracellular antigens**					
TNFα	Mab11	IgG1	Alexa fluor 700	Direct	Biolegend
IFNγ	P2G10	IgG1	PE	Direct	BD Biosciences

^A^ Rat-anti-mouse anti-IgG2a-PerCPfluor710, eBiosscience.

**Table 2:** Overview of the most confident hits of mass spectra obtained from the analysis of MHC-I-bound peptides of PRRSV-infected PAMs matched with the PRRSV genome.

Peptide	Sequence	PRRSV Protein Origin	Length (Amino Acids)
1	SVVFPLARM	NSP1α	9
2	LVKVAEVLYR	NSP1α	10
3	RLQINGIR	NSP1β	8
4	LDKMWDRV	NSP2	8
5	LALEQRQL	NSP2	8
6	VISESGDLI	NSP4	9
7	DIKLSPAII	NSP4	9
8	SQALSTYCF	NSP5	9
9	VEKLKRII	NSP8	8
10	QGFVLPGVL	NSP9	9
11	GRCLEADL	NSP9	8
12	LLEIQPML	NSP9	8
13	VITDKPSFL	NSP9	9

**Table 3:** SLA-I low-resolution haplotypes (Lr-Hp) of PAMs used for peptide isolation and PBMCs for in vitro restimulation.

Pig	SLA-1	SLA-3	SLA-2	Lr-Hp
PAM ^1^	12XX, 13XX	05XX	10XX	35.0
	07XX, 08XX	04XX	06XX	24.0 mod
Gilt16 ^2^	12XX, 13XX	05XX	10XX	35.0
	02XX, 18:01	01XX	11XX	57.0
Gilt24 ^2^	12XX, 13XX	05XX	10XX	35.0
	02XX, 07XX	04XX	02XX	2.0
Piglet55 ^2^	13XX	04XX	06XX	24.0 mod
	08XX	05XX	blank	49.0
Piglet62 ^2^	blank 11:03	04XX 05XX	06XX 16:02	24.0 59.0

^1^ PAMs used for isolation of MHC-I peptides; ^2^ PBMCs used for restimulation with synthetic peptides. mod, modified

## Data Availability

The mass spectrometry proteomics data have been deposited to the ProteomeXchange Consortium via the PRIDE [45] partner repository with the dataset identifier PXD035499 and 10.6019/PXD035499.
